# The Influence of PAR 1 and Endothelin 1 on the Course of Specific Kidney Diseases

**DOI:** 10.3390/jcm15010221

**Published:** 2025-12-27

**Authors:** Maciej Szymczak, Marcelina Żabińska, Katarzyna Kościelska-Kasprzak, Dorota Bartoszek, Harald Heidecke, Kai Schulze-Forster, Łucja Janek, Krzysztof Kujawa, Jakub Wronowicz, Karolina Marek-Bukowiec, Tomasz Gołębiowski, Mirosław Banasik

**Affiliations:** 1 Clinical Department of Nephrology, Transplantation Medicine and Internal Diseases, Institute of Internal Diseases, Wroclaw Medical University, 50-556 Wroclaw, Poland; karolina.marek-bukowiec@umw.edu.pl (K.M.-B.); tomasz.golebiowski@umw.edu.pl (T.G.); miroslaw.banasik@umw.edu.pl (M.B.); 2 Department of Non-Procedural Clinical Sciences, Faculty of Medicine, Wrocław University of Science and Technology, 50-370 Wroclaw, Poland; marcelina.zabinska@pwr.edu.pl (M.Ż.); katarzyna.koscielska-kasprzak@pwr.edu.pl (K.K.-K.); dorota.bartoszek@pwr.edu.pl (D.B.); 3 CellTrend Gmbh, Im Biotechnologiepark 3 TGZ II, 14943 Luckenwalde, Germany; heidecke@celltrend.de (H.H.); schufo@celltrend.de (K.S.-F.); 4 Statistical Analysis Center, Wroclaw Medical University, 50-368 Wroclaw, Poland; lucja.janek@umw.edu.pl (Ł.J.); krzysztof.kujawa@umw.edu.pl (K.K.); jakub.wronowicz@umw.edu.pl (J.W.)

**Keywords:** glomerulonephritis, protease-activated receptor 1, endothelin 1, chronic kidney disease, hemodialysis

## Abstract

**Background**: PAR 1 (protease-activated receptor 1) and endothelin 1 are biomarkers that could be of significance in kidney diseases. **Methods**: We measured the plasma levels of PAR1 and endothelin 1 in patients with membranous nephropathy (*n* = 19), focal and segmental glomerulosclerosis (FSGS) (*n* = 30), systemic lupus erythematosus (SLE) (*n* = 22), IgA nephropathy (*n* = 16), mesangial proliferative (non-IgA) glomerulonephritis (*n* = 7), chronic kidney disease (CKD) (*n* = 27), and hemodialysis (*n* = 26), as well as a healthy control group (*n* = 22). Then, for two years, we tracked the patients’ clinical progress (creatinine, total protein, and albumin levels) and compared the outcomes with their initial PAR 1 and endothelin 1 levels. Moreover, we checked the correlations between PAR 1 and endothelin 1 and the results of anti-PAR1 and anti-ETAR (endothelin A receptor) evaluations. **Results**: Membranous nephropathy, FSGS, IgA nephropathy, CKD, and hemodialysis patients had higher PAR 1 levels than the control group. PAR 1 correlated with total protein, albumin in SLE, total protein in IgA nephropathy, and creatinine in CKD. Endothelin 1 correlated with albumin in membranous nephropathy, total protein, albumin, creatinine in FSGS, total protein in IgA nephropathy, total protein, and albumin in CKD. PAR 1 correlated with anti-PAR 1 in FSGS. Anti-ETAR correlated with anti-PAR 1 in membranous nephropathy, FSGS, and IgA nephropathy. **Conclusions**: PAR 1 levels are elevated in some kidney diseases compared to the healthy population. Both PAR 1 and endothelin 1 are supposed to be related to the clinical course of specific kidney diseases.

## 1. Introduction

Protease-activated receptor type 1 (PAR 1) is a receptor from the G protein-coupled receptor family [1] that attaches to thrombin [2]. PAR 1 is involved in interactions with serine proteases [3], trypsin [4], neutrophil elastase [5], plasmin [6], and kallikrein [7]. PAR 1 is found on podocytes, the lining of blood vessels, and kidney epithelial cells [8], and it is activated by both thrombin and factor Xa [9].

Endothelin 1 is a vasoactive protein that modulates vascular function and influences endothelial cells [10]. Endothelin 1 is considered a ‘profibrotic cytokine’ [11] and influences the development of kidney inflammation [12]. Endothelin 1 is present in every cell in the body, but its concentration in the kidneys is high, especially in the case of kidney diseases [13].

Endothelin 1 connects with two receptors: endothelin receptor A (ETAR) and endothelin receptor B (ETBR) [14]. These receptors belong to the G protein-coupled receptor family [15].

The influence of both PAR1 and endothelin 1 on processes connected to clotting, and the fact that the PAR1 and endothelin 1 receptors belong to the same family of receptors (G protein-coupled), are the reasons why we have decided to check the levels of these biomarkers together. 

Our null hypotheses were to check differences between PAR 1 and endothelin 1 levels in specific kidney diseases, the significance of basic PAR 1 and endothelin 1 levels, their correlations with each other, and the correlations of these biomarkers with clinical markers of specific diseases (total protein, albumin, and creatinine) using prospective observation. We documented the health results of patients over a span of two years after the assessment of PAR 1 and endothelin 1 levels. This way, we can verify if these proteins could eventually be recognized as possible prognostic factors in specific kidney diseases.

Moreover, some of our patients took over on our previous projects with anti-PAR 1 [16] and anti-ETAR [17] antibodies. Materials from these patients were taken on the same day as for previous projects for the same venepuncture, so we planned to check correlations between PAR 1, endothelin 1, anti-PAR 1, and anti-ETAR in these cases.

What is the rationale for evaluating PAR 1 and endothelin 1 in kidney diseases?

The activation of PAR 1 leads to vasoconstriction [18], reduces renin levels [19], contributes to platelet activation and clumping [20], and aids in the migration of endothelial cells [21].

Consequently, blocking PAR 1 protects the endothelium and enhances recovery from vascular injury [22]. PAR 1 insufficiency prevents the development of diabetes caused by streptozotocin in studies involving mice [23], while certain findings suggest that PAR 1 inhibitors mitigate kidney toxicity related to fibrosis, tubule inflammation, and mitochondrial issues [24]. In contrast, stimulating PAR 1 in human proximal tubule epithelial cells decreases the amounts of transforming growth factor-beta (TGF-β) and minimizes the generation of interleukin 6 (IL-6) and IL-8, which are induced by tumor necrosis factor-alpha (TNF-α) [25]. PAR 1 contributes to the progression of kidney inflammation in cases of crescentic glomerulonephritis [26]; conversely, triggering PAR 1 in mice leads to a model similar to focal and segmental glomerulosclerosis (FSGS) [27]. The stimulation or inhibition of PAR 1 resulted in reduced kidney functionality in a study involving rats, whereas typical PAR 1 operation promoted the optimal preservation of kidney efficiency [28]. However, not all outcomes seen in studies involving rats can be applied to humans [29].

Calcium fluctuations that are mediated by PAR 1 have been demonstrated to affect the growth of mesangial cells [30]. PAR 1 was also found to be a factor involved in chronic pain development in systemic lupus erythematosus [31] and kidney allograft vasculopathy activation [32].

TNF-alpha, interleukin 1-beta, and IL-6 induce endothelin 1 production, leading to positive feedback and the development of inflammation [33]. NF-kappa B is induced by endothelin 1 [34]. On the other hand, endothelin 1 can suppress IL-12 production and dendritic cell signalling and survival through the endothelin A receptor. This contrary effect appears after the activation of the endothelin B receptor [35]. Studies on specific diseases revealed the role of endothelin 1 in the development of hypertensive kidney disease [36], IgA nephropathy [37,38], focal and segmental glomerulosclerosis [39], diabetic nephropathy [40], acute kidney injury [41], ischemia–reperfusion injury [41], polycystic kidney disease [42], sickle cell nephropathy [43], and systemic sclerosis [44].

There is a possible therapeutic intervention involving the endothelin 1 system through an endothelin 1 antagonist. Unfortunately, the first established endothelin 1 antagonist, bosentan, was withdrawn from usage because of hepatotoxic effects [45]. Nevertheless, later drugs from this group were developed, and some clinical trials were performed for kidney diseases in the past few years. The ZENITH-CKD (CKD—chronic kidney disease) study revealed the positive effect of zibotentan in terms of albuminuria reduction when used with dapagliflozin, compared to dapagliflozin with a placebo [46]. The diuretic effect of dapagliflozin is equal to the fluid retention associated with zibotentan [47].

Sparsentan (both an endothelin A receptor and an angiotensin II type 1 receptor 1 antagonist) was better in terms of proteinuria reduction compared to irbesartan in focal and segmental glomerulosclerosis patients (DUET study) [48].

Sparsentan was also better than irbesartan in reducing protein in IgA nephropathy (PROTECT trial) [49]. The novel endothelin A receptor antagonist atrasentan was also effective in reducing proteinuria in IgA nephropathy (ALIGN trial) [50].

## 2. Materials and Methods

Individuals displaying the initial indications of glomerular disease or experiencing a recurrence and who attended our clinic from 2013 to 2021 were included in this study before beginning immunosuppressive treatments (including cyclophosphamide and steroid pulses) and prior to the commencement of ACE inhibitor therapy. At the moment of enrollment or within the six months leading up to it, none of the participants diagnosed with glomerular diseases took any medications. Because of the structure of this study, randomization was not utilized. This research only included patients who had a histopathological diagnosis of IgA nephropathy, non-IgA mesangial proliferative glomerulopathy, primary focal and segmental glomerulosclerosis, and membranous nephropathy. In addition, patients with glomerulonephritis needed to show proteinuria (at least 60 mg per day) during their most recent urine test (taken one or two days before sample collection) to be eligible for this study.

**Figure 1:**
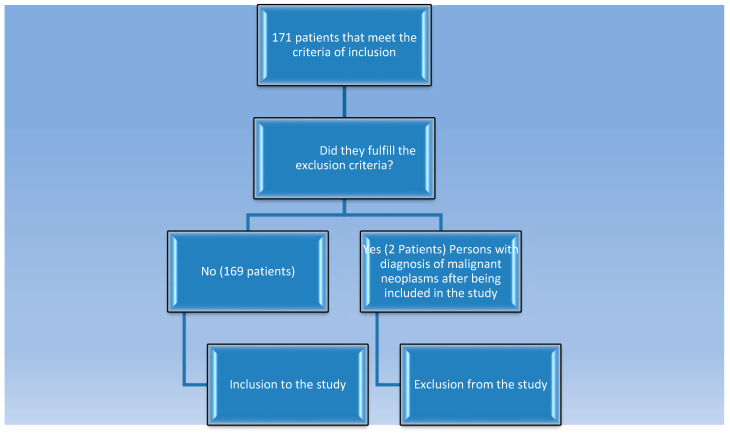
A diagram illustrating the process of participating in the project, which was followed by the study participants.

Moreover, we recruited groups of patients with chronic kidney disease (eGFR-estimated glomerular filtration rate below 60 mL/min, but before starting dialysis) and people on chronic hemodialysis (hemodialyzed for at least 3 months). This study excluded participants who are presently experiencing infections, as well as those who have received kidney transplantation, or anyone with a history of cancer, whether in the past or present. All of these conditions can affect the immune system and alter the results of biomarker levels. The control group consisted of 22 healthy individuals of Caucasian origin who met specific standards: they possessed neither a personal nor a familial history of kidney problems, autoimmune disorders, or cancer-related problems. The concentrations of C-reactive protein (CRP) were below 5 mg/dL, their creatinine levels did not exceed 1.3 mg/dL, and they exhibited no signs of proteinuria or erythrocyturia. The controls used for comparison included employees from our clinic and their friends, and their selection was made in a random and sequential manner without further criteria beyond those previously stated. A diagram illustrating the process followed by the study participants is presented in Figure 1.

All individuals involved in the research were from Poland, and each completed an informed consent document before any materials were collected. This study was granted approval by the Bioethics Committee at Wrocław Medical University, with the identification numbers KB-546 in 2012 and KB-221 in 2023. This research received approval from the Bioethics Committee at Wrocław Medical University, identified by the numbers KB-221/2023 and KB-546/2012. Plasma samples were obtained from a group of 169 healthy controls alongside patients diagnosed with the following conditions: membranous glomerulonephritis (*n* = 19), focal and segmental glomerulosclerosis (FSGS) (*n* = 30), systemic lupus erythematosus with kidney involvement (SLE) (*n* = 22), IgA nephropathy (*n* = 16), non-IgA mesangial proliferative glomerulonephritis (*n* = 7), chronic kidney disease (*n* = 27), and hemodialysis (*n* = 26), as well as a control group (*n* = 22).

These are patients recruited for plasma PAR 1 and plasma endothelin 1 evaluation. Additionally, some of these patients were recruited for our previous studies [16,17], and we used the anti-PAR 1 and anti-ETAR results from these patients to perform correlation studies between PAR 1, endothelin 1, anti-PAR 1, and anti-ETAR: membranous nephropathy (*n* = 17), focal and segmental glomerulosclerosis (*n* = 25), SLE (*n* = 17), nephropathy IgA (*n* = 14), and mesangial proliferative (non-IgA) glomerulonephritis (*n* = 6).

A secondary collection tube was employed to acquire an additional 2.7 mL of blood from each participant without requiring another venipuncture, thereby supplementing the blood collected for standard laboratory tests. Patients were not required to skip breakfast on the morning of the blood sample collection. To mitigate any bias introduced by differences in storage time before freezing, the samples were centrifuged exactly ten minutes post-collection. Blood samples were not gathered when the temperature surpassed 28 °C, in accordance with our laboratory’s protocols. After centrifuging the blood at 1500× *g* for ten minutes, the serum was separated and stored at −80 °C.

The assessment of PAR1 was carried out using an ELISA kit (Human PAR1/Thrombin Receptor ELISA Kit, Abcam, Cambridge, United Kingdom) following the instructions provided by the manufacturer. Removable 8-well strips were labelled. Subsequently, we introduced 100 μL of either the standard or the sample into the designated wells. The wells were then covered and incubated for 2.5 h at room temperature with gentle shaking. The solution was discarded, and the wells were washed four times with 1× Wash Solution. Each well was filled with Wash Buffer (300 μL) using a multi-channel pipette or an auto-washer. Liquid was thoroughly removed at each stage. After the final wash, any residual Wash Buffer was eliminated by aspiration.

The plate was turned upside down and pressed against clean paper towels. Then, 100 μL of the 1 X biotinylated antibody, which had been prepared, was introduced into each well. Following this, the mixture was incubated for 1 h at room temperature with gentle shaking. The solution was then discarded. The washing step was repeated. Subsequently, 100 μL of the prepared Streptavidin solution was added to each well. Incubation for 45 min at room temperature with gentle shaking was then carried out.

Subsequently, the solution was discarded. The washing process was repeated. First, 100 μL of the TMB (3, 3′, 5,5′-tetramethylbenzidine) Substrate Reagent was introduced into each well. Following this, incubation occurred for 30 min at room temperature in the dark with gentle shaking. Then, 50 μL of Stop Solution was added to each well. The reading at 450 nm was conducted immediately. The detection limit ranged from 3.125 to 200 pg/mL. The intra-assay coefficient of variation was 8%, while the inter-assay coefficient of variation was 9%.

Endothelin 1 evaluation proceeded with usage of the ELISA kit (Endothelin 1 ELISA Kit, Abcam, Cambridge, United Kingdom). First, 100 µL of the standards and samples were added to the appropriate wells. The plate was sealed and incubated for 1 h at room temperature. The contents of the wells were emptied and washed by adding 400 µL of 1 × Wash Buffer to every well. Washing was repeated 4 more times, for a total of 5 washes. After the final wash, the wells were emptied, and the plate was firmly tapped on a lint-free paper towel to remove any remaining wash buffer. Then, 100 μL of the Endothelin 1 antibody was added into each well. The plate was sealed. The plate was incubated at room temperature for 30 min. Then, washing proceeded as described earlier. A total of 100 μL of the TMB Substrate solution was added to every well. Incubation at room temperature for 30 min was then carried out. After, 100 μL of Stop Solution was added to each well. The plate was read immediately. The reading was performed at 450 nm, with a correction of 580 nm. The detection limit was 0.78–100 pg/mL. The intra-assay coefficient of variation was 9%, and the inter-assay coefficient of variation was 9%. The evaluations of the anti-PAR 1 and anti-ETAR antibodies were performed as described in our previous works [16,17].

Samples for all evaluations were analyzed in duplicate.

Clinical data were collected regarding serum levels of creatinine, the estimated glomerular filtration rate (eGFR), calculated using the MDRD formula, which stands for Modification of Diet in Renal Disease, blood urea nitrogen (BUN), the identification of protein in urine, the urine albumin per creatinine ratio, serum protein (total) levels, and serum albumin concentrations at the start of the observation period, as well as at one month, three months, six months, one year, and two years. The sex and age of the participants were also recorded.

The levels of PAR 1, endothelin 1, anti-PAR 1, anti-ETAR, eGFR, BUN, serum creatinine, serum albumin, serum protein (total), proteinuria, and the albumin per creatinine ratio, as well as sex and age, were analyzed and compared between the groups of patients with kidney diseases and the control group through the application of the Kruskal–Wallis test, Dunn’s test with Bonferroni corrections, or ANOVA. Adjustments for age and sex were implemented.

To evaluate the relationships between numerical variables, either Pearson’s or Spearman’s correlation coefficients were utilized, following a Shapiro–Wilk test to examine the data distribution. A *t*-test was conducted to determine the significance of the correlations, with a *p*-value of 0.05 or lower deemed significant (95% confidence interval). There were no primary sample calculations for this study. Adjustments were made to *p*-values for multiple comparisons with Bonferroni correction. This correlation examination explored the relationship between PAR 1 levels, endothelin 1 levels, and clinical information.

The concentrations of ANAs (antinuclear antibodies) and ds DNA (anti-double-stranded deoxyribonucleic acid antibodies) in patients with lupus nephritis were then assessed in relation to the levels of PAR 1 and endothelin 1.

Utilizing the baseline concentrations of PAR 1 and endothelin 1 from each category, Spearman’s correlation was employed to analyze the fluctuations in clinical indicators (including BUN, eGFR, serum creatinine, serum albumin, and serum protein (total) levels) during a two-year follow-up period. The assessment included examining the coefficient of variation, the standard deviation, the statistical range, and the trajectory of antibody concentrations throughout the project duration. Data analyses were conducted using STATISTICA 13.

## 3. Results

### 3.1. Patients’ Clinical Data

The clinical data from the patients are presented in Table 1 and Table 2. The groups were similar in terms of most of the data, other than what is mentioned below.

Patients diagnosed with mesangial proliferative (non-IgA) glomerulonephritis were found to be younger in comparison to the other groups (*p* = 0.01).

Membranous nephropathy and focal and segmental glomerulosclerosis patients exhibited higher levels of proteinuria when compared to the other groups (*p* = 0.01). The control group demonstrated lower proteinuria levels than the other groups, as patients in the control group exhibited no proteinuria (*p* = 0.01).

There was a greater number of males in the membranous nephropathy and focal and segmental glomerulosclerosis groups in contrast to the lupus nephritis and mesangial proliferative (non-IgA) glomerulonephritis groups (*p* = 0.004).

### 3.2. PAR 1 Evaluation Results

The median plasma levels of PAR 1 were recorded at 69.07 (27.19–210) pg/mL for the membranous nephropathy cohort; 70.46 (43.64–210) pg/mL for the focal and segmental glomerulosclerosis cohort; 60.2 (35.33–210) pg/mL for the systemic lupus erythematosus cohort; 98.43 (36.2–210) pg/mL for the IgA nephropathy cohort; 53.79 (39.48–143.46) pg/mL for the mesangial proliferative (non-IgA) glomerulonephritis cohort; 106.36 (35.39–210) pg/mL for the hemodialysis cohort; 99.78 (34.08–210) pg/mL for the chronic kidney disease cohort; and 33.41 (20.8–108.52) pg/mL for the control group.

Plasma PAR 1 levels in the focal and segmental glomerulosclerosis, IgA nephropathy, hemodialysis, and chronic kidney disease cohorts were higher compared to the control group (*p* < 0.001 for all the above-mentioned groups).

**Figure 2:**
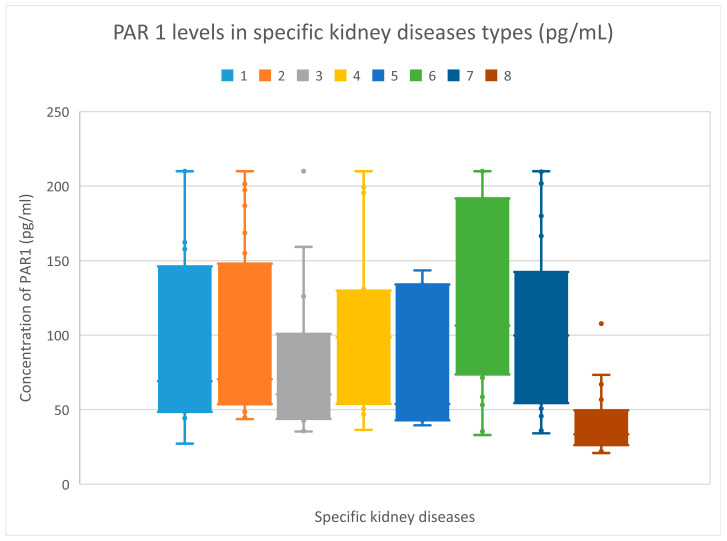
PAR 1 levels in specific renal disorders (pg/mL). The bars with colors indicated by the numbers 1–8 show PAR 1 levels in patient groups 1–8. The *y*-axis displays the PAR 1 concentration (U/mL). Patients are represented by dots. 1—membranous glomerulonephritis; 2—focal and segmental glomerulosclerosis; 3—systemic lupus erythematosus; 4—IgA nephropathy; 5—mesangial proliferative (non-IgA) glomerulopathy; 6—hemodialysis; 7—chronic kidney disease; 8—control group.

The plasma levels of PAR 1 in specific cohorts are displayed in Figure 2.

### 3.3. Endothelin 1 Evaluation Results

The median plasma levels of endothelin 1 levels were recorded at 0.63 (0–53) pg/mL for the membranous nephropathy cohort; 0.57 (0–53) pg/mL for the focal and segmental glomerulosclerosis cohort; 0.50 (0–10.94) pg/mL for the systemic lupus erythematosus cohort; 0.44 (0–20.03) pg/mL for the IgA nephropathy cohort; 0.24 (0–4.49) pg/mL for the mesangial proliferative (non-IgA) glomerulonephritis cohort; 2.01 (0.32–30.21) pg/mL for the hemodialysis cohort; 1.18 (0.5–44.78) pg/mL for the chronic kidney disease cohort; and 1.46 (0.32–53) pg/mL for the control group.

Plasma endothelin 1 levels do not differ statistically between the groups.

**Figure 3:**
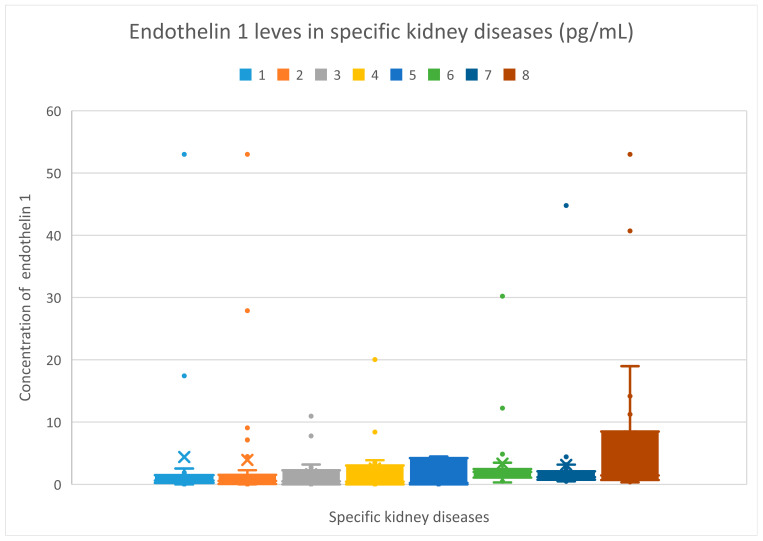
Endothelin 1 levels (pg/mL) in specific kidney diseases. The bars with colors indicated by the numbers 1–8 show endothelin 1 levels in patient groups 1–8. The *y*-axis displays the endothelin 1 concentration (U/mL). Patients are represented by dots. 1—membranous glomerulonephritis; 2—focal and segmental glomerulosclerosis; 3—systemic lupus erythematosus; 4—IgA nephropathy; 5—mesangial proliferative (non-IgA) glomerulopathy; 6—hemodialysis; 7—chronic kidney disease; 8—control group.

The values of plasma endothelin 1 levels in specific cohorts are displayed in Figure 3.

### 3.4. Relationship Between Clinical Patient Data and PAR 1 Levels

We found statistically significant correlations between initial plasma PAR 1 levels and the clinical data, both initially and when monitored over time.

**Figure 4:**
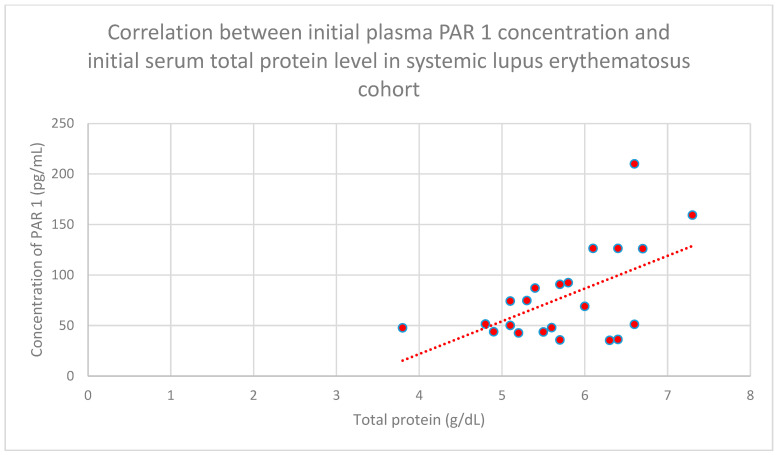
Relationship between initial plasma PAR 1 levels and initial serum total protein levels in the systemic lupus erythematosus cohort (*p* = 0.0056; r = 0.41). Specific patients are shown as red dots. The line indicating the trend is depicted by the red dotted line.

We found a relationship between initial plasma PAR 1 levels and initial serum total protein levels in the systemic lupus erythematosus group (*n* = 22; *p* = 0.0056; r = 0.41) (Figure 4).

**Figure 5:**
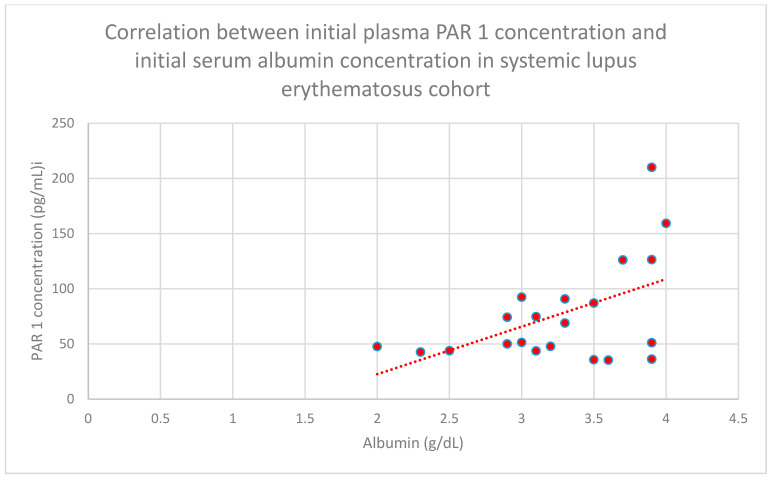
Relationship between initial plasma PAR 1 concentrations and initial serum albumin concentrations in the systemic lupus erythematosus cohort (*p* = 0.05; r = 0.42). Specific patients are shown as red dots. The line indicating the trend is depicted by the red dotted line.

In addition, a statistically significant correlation was observed between the initial plasma PAR 1 concentration and the initial serum albumin concentration in the systemic lupus erythematosus cohort (*n* = 22; *p* = 0.05; r = 0.42) (Figure 5).

**Figure 6:**
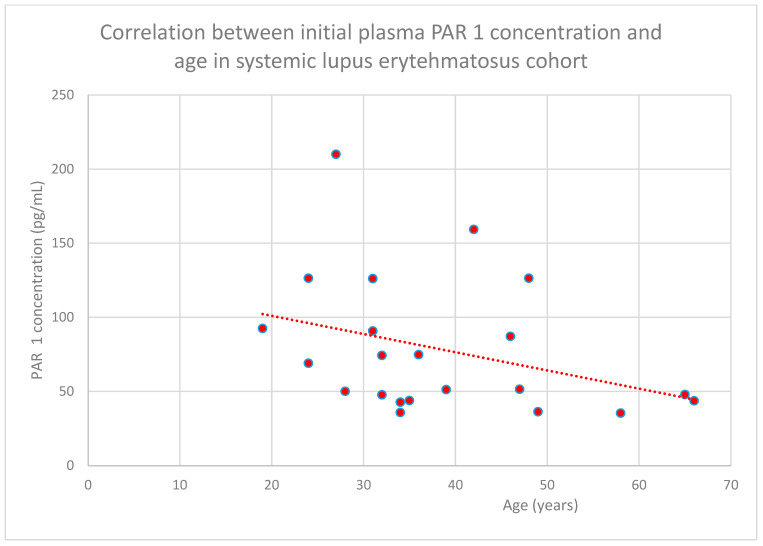
Relationship between initial plasma PAR 1 concentrations and age in the systemic lupus erythematosus cohort (*p* = 0.04 r = −0.43). Specific patients are shown as red dots. The line indicating the trend is depicted by the red dotted line.

The negative relationship between the initial plasma PAR 1 concentration and age in the systemic lupus erythematosus cohort also reached statistical significance (*n* = 22; *p* = 0.04; r = −0.43) (Figure 6).

**Figure 7:**
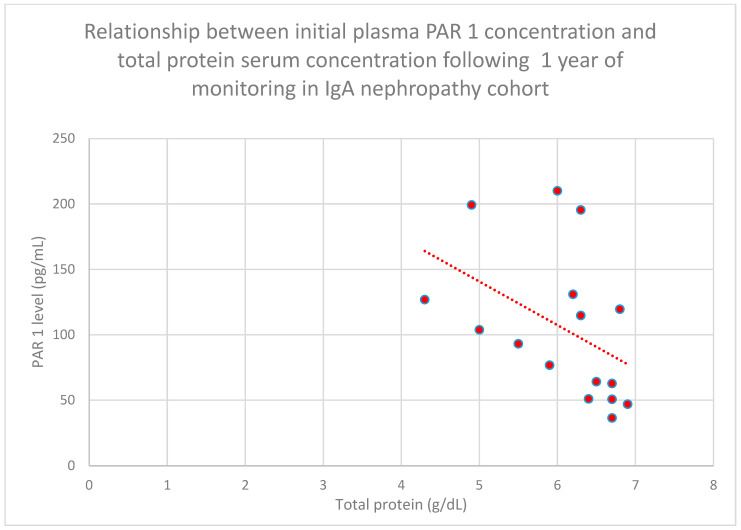
Relationship between initial plasma PAR 1 concentrations and initial serum total protein concentrations following 1 year of monitoring in the IgA nephropathy cohort (*p* = 0.01; r = −0.6). Specific patients are shown as red dots. The line indicating the trend is depicted by the red dotted line.

The relationship between initial plasma PAR 1 concentrations and serum total protein concentrations after 1 year of monitoring in the IgA nephropathy cohort was also negative (*n* = 16; *p* = 0.01; r = −0.6) (Figure 7).

**Figure 8:**
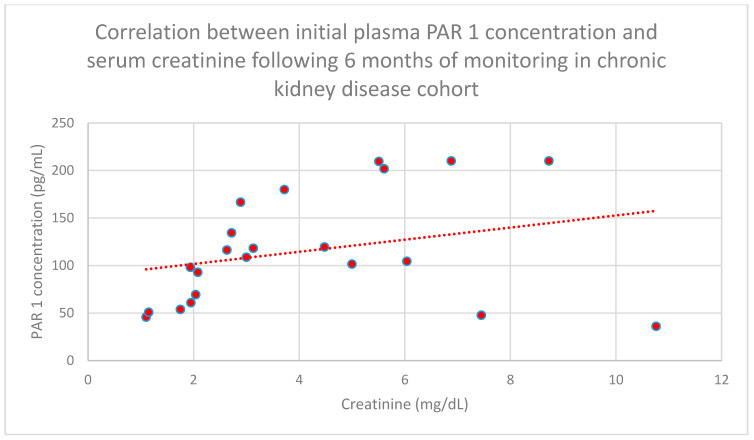
Relationship between initial plasma PAR 1 concentrations and initial serum creatinine concentrations following 6 months of monitoring in the chronic kidney disease cohort (*p* = 0.05; r = 0.42). Specific patients are shown as red dots. The line indicating the trend is depicted by the red dotted line.

**Figure 9:**
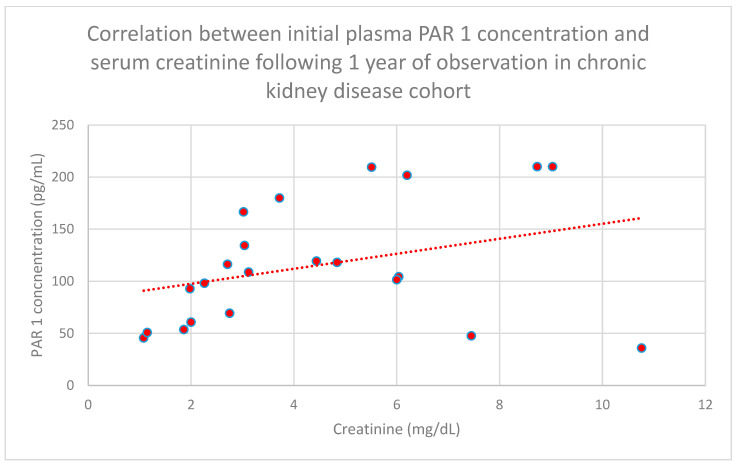
Relationship between initial plasma PAR 1 concentrations and initial serum creatinine concentrations following 1 year of monitoring in the chronic kidney disease cohort (*p* = 0.04; r = 0.43). Specific patients are shown as red dots. The line indicating the trend is depicted by the red dotted line.

**Figure 10:**
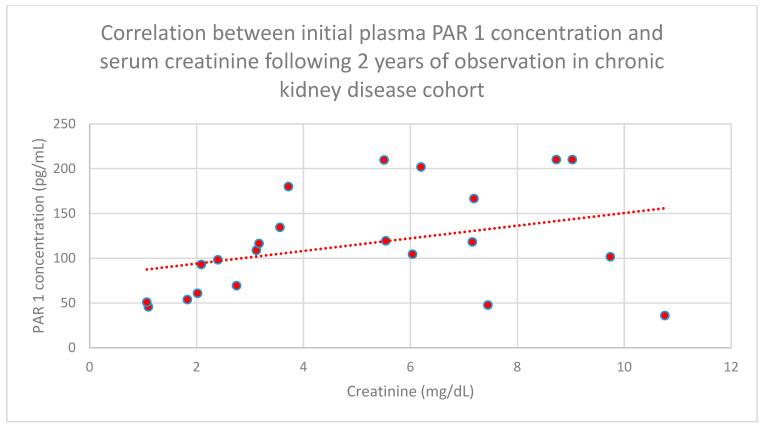
Relationship between initial plasma PAR 1 concentrations and serum creatinine concentrations following 2 years of monitoring in the chronic kidney disease cohort (*p* = 0.05; r = 0.41). Specific patients are shown as red dots. The line indicating the trend is depicted by the red dotted line.

We also found a relationship between initial plasma PAR 1 concentrations and serum creatinine concentrations following 6 months (*n* = 27; *p* = 0.05; r = 0.42) (Figure 8), 1 year (*n* = 27; *p* = 0.04; r = 0.43) (Figure 9), and 2 years (*n* = 27; *p* = 0.05; r = 0.41) (Figure 10) of monitoring in the chronic kidney disease cohort.

### 3.5. Relationship Between Patients’ Clinical Data and Endothelin 1 Levels

**Figure 11:**
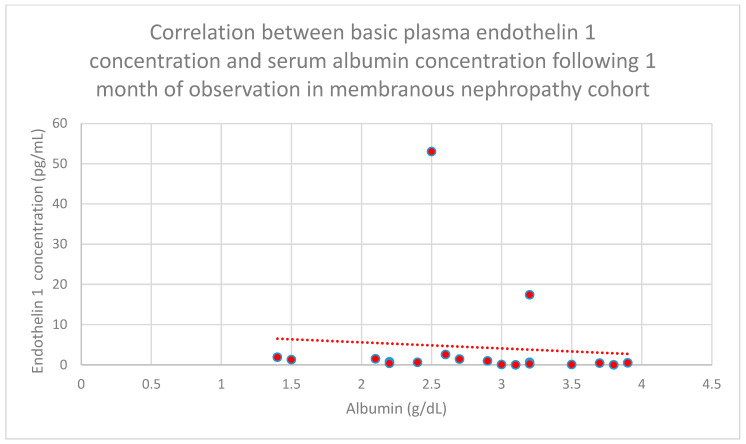
Relationship between initial plasma endothelin 1 concentrations and albumin concentrations in serum after 1 month of tracking in the membranous nephropathy cohort (*p* = 0.05; r = −0.44). Specific patients are shown as red dots. The line indicating the trend is depicted by the red dotted line.

**Figure 12:**
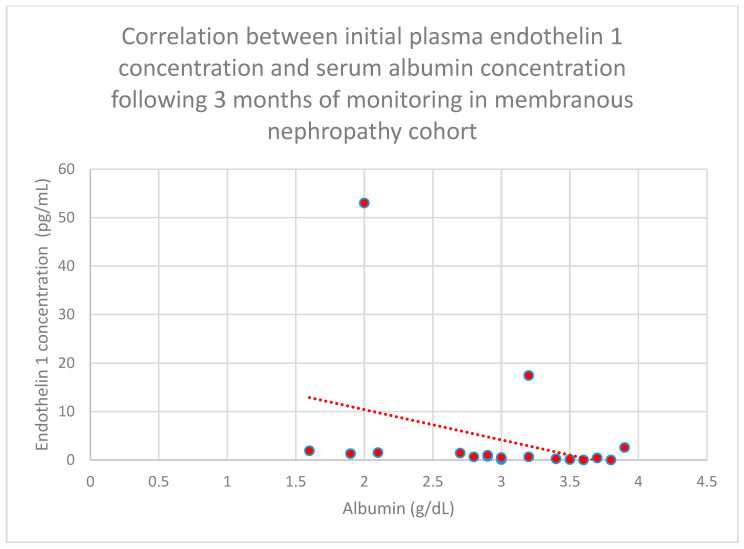
Relationship between initial plasma endothelin 1 concentrations and serum albumin concentrations following 3 months of monitoring in the membranous nephropathy cohort (*p* = 0.01; r = −0.54). Specific patients are shown as red dots. The line indicating the trend is depicted by the red dotted line.

**Figure 13:**
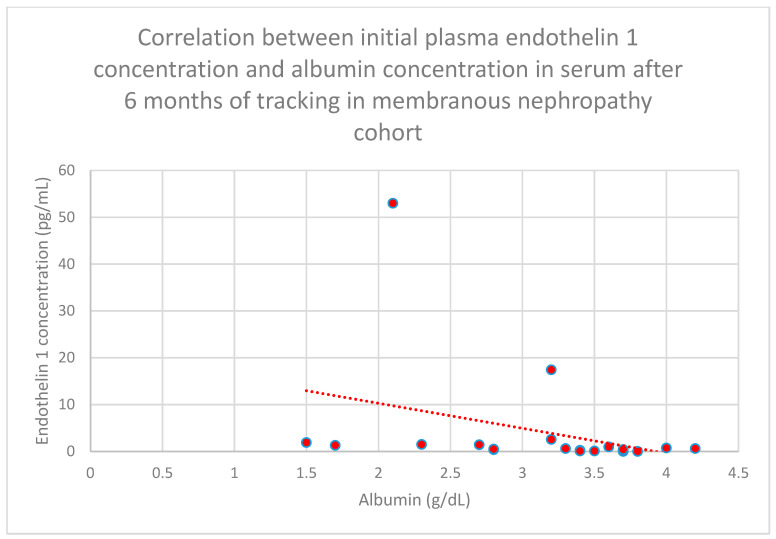
Relationship between initial plasma endothelin 1 concentrations and serum albumin concentrations after 6 months of tracking in the membranous nephropathy cohort (*p* = 0.007; r = −0.6). Specific patients are shown as red dots. The line indicating the trend is depicted by the red dotted line.

**Figure 14:**
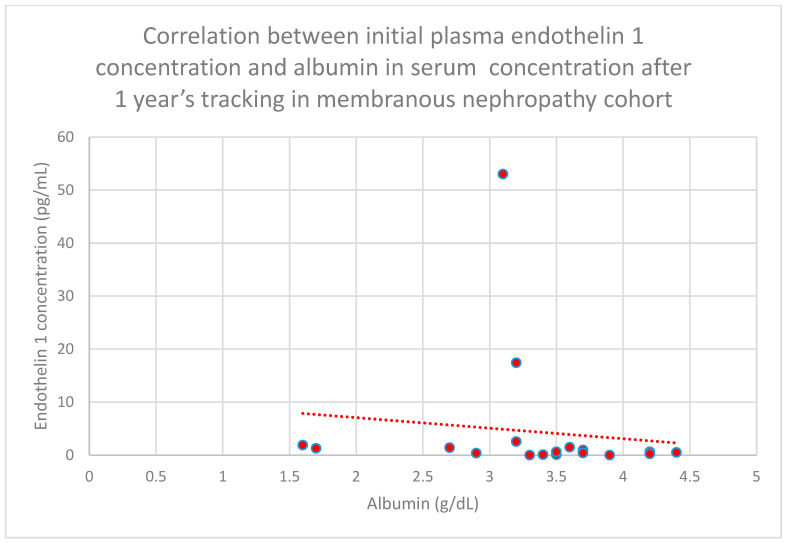
Relationship between initial plasma endothelin 1 concentrations and serum albumin concentrations after 1 year of tracking in the membranous nephropathy cohort (*p* = 0.04; r = −0.46). Specific patients are shown as red dots. The line indicating the trend is depicted by the red dotted line.

Relationships between initial plasma endothelin 1 concentrations and serum albumin concentrations following 1 month (*n* = 19; *p* = 0.05; r = −0.44) (Figure 11), 3 months (*n* = 19; *p* = 0.01; r = −0.54) (Figure 12), 6 months (*n* = 19; *p* = 0.007; r = −0.6) (Figure 13), and 1 year (*n* = 19; *p* = 0.04; r = −0.46) (Figure 14) of monitoring in the membranous nephropathy cohort were statistically significantly negative.

**Figure 15:**
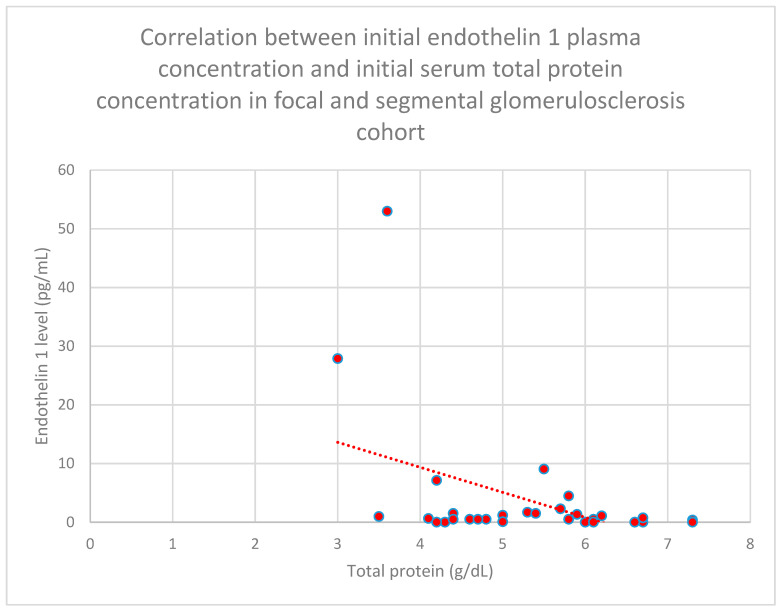
Relationship between initial plasma endothelin 1 concentrations and initial total serum protein concentrations in the focal and segmental glomerulosclerosis cohort (*p* = 0.03; r = −0.38). Specific patients are shown as red dots. The line indicating the trend is depicted by the red dotted line.

The correlation between initial plasma endothelin 1 concentrations and initial serum total protein concentrations in the focal and segmental glomerulonephritis cohort was negative (*n* = 30; *p* = 0.03; r = −0.38) (Figure 15).

**Figure 16:**
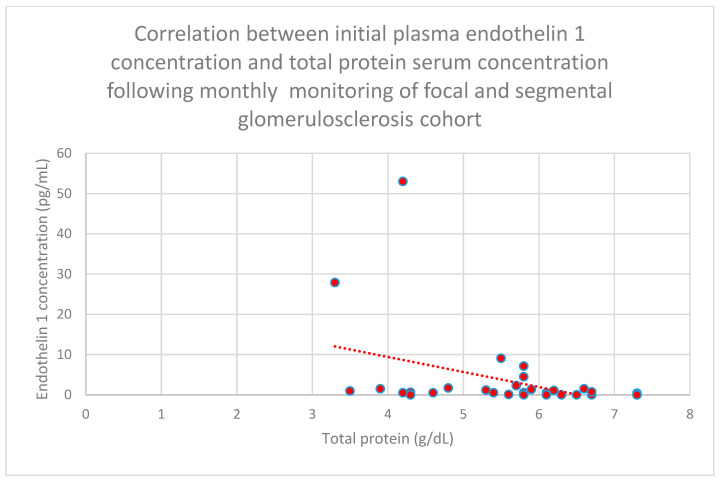
Relationship between initial plasma endothelin 1 concentrations and total serum protein concentrations following monthly monitoring of the focal and segmental glomerulosclerosis cohort (*p* = 0.02; r = −0.41). Specific patients are shown as red dots. The line indicating the trend is depicted by the red dotted line.

**Figure 17:**
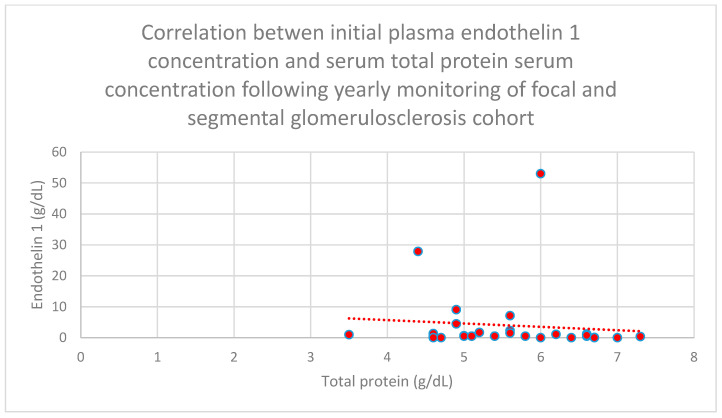
Relationship between initial plasma endothelin 1 concentrations and serum total protein concentrations following yearly monitoring of the focal and segmental glomerulosclerosis cohort (*p* = 0.04; r = −0.37). Specific patients are shown as red dots. The line indicating the trend is depicted by the red dotted line.

**Figure 18:**
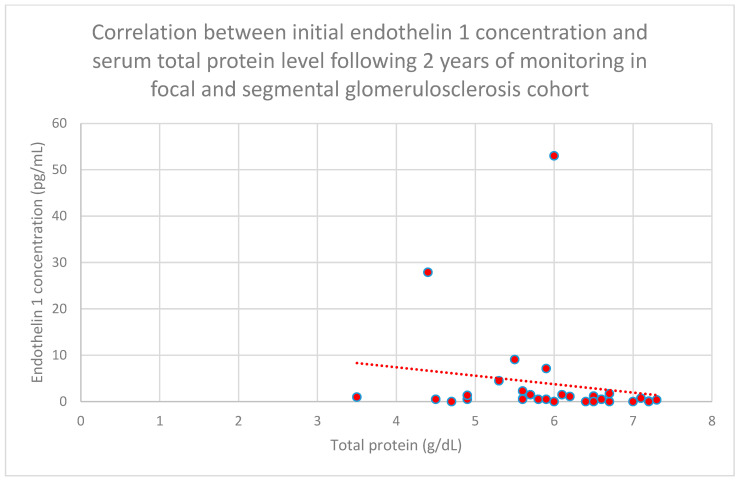
Relationship between initial plasma endothelin 1 concentrations and serum total protein concentrations following 2 years of tracking in the focal and segmental glomerulonephritis cohort (*p* = 0.03; r = −0.39). Specific persons are shown as red dots. The line indicating the trend is depicted by the red dotted line.

Relationships between initial plasma endothelin 1 concentrations and total serum protein concentrations after 1 month (*n* = 30; *p* = 0.02; r = −0.41) (Figure 16), 1 year (*n* = 30; *p* = 0.04; r = −0.37) (Figure 17), and 2 years (*n* = 30; *p* = 0.03; r = −0.39) (Figure 18) of monitoring in the focal and segmental glomerulosclerosis cohort were negative and statistically significant.

**Figure 19:**
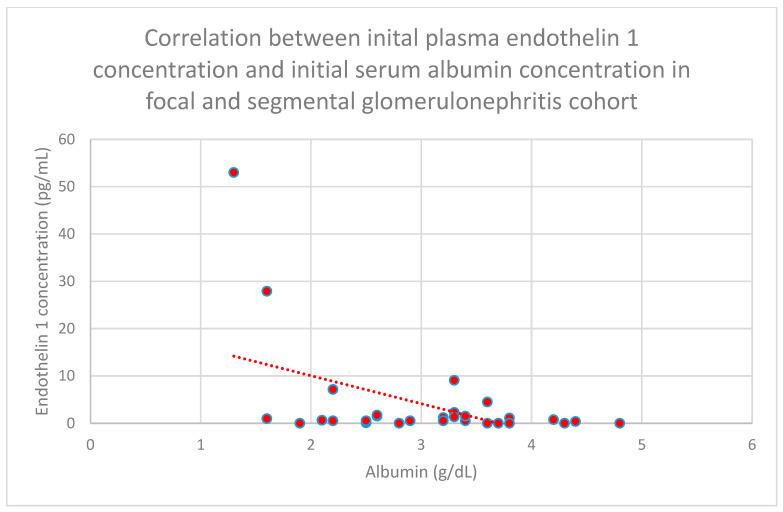
Relationship between initial plasma endothelin 1 concentrations and initial serum albumin concentrations in the focal and segmental glomerulonephritis cohort (*p* = 0.05; r = −0.36). Specific persons are shown as red dots. The line indicating the trend is depicted by the red dotted line.

**Figure 20:**
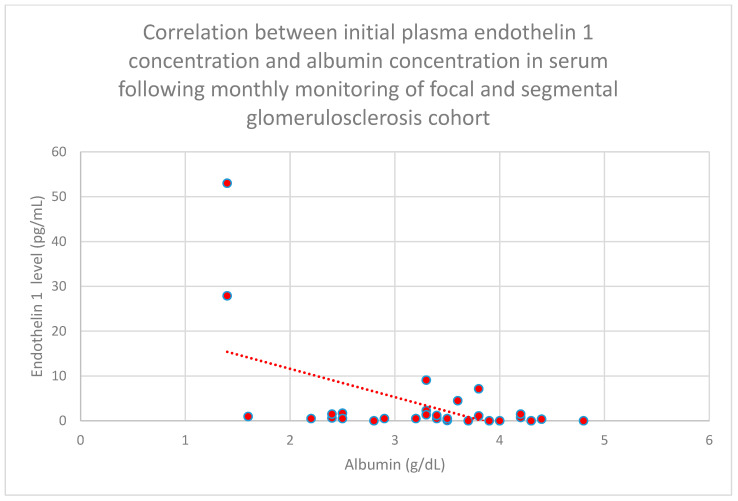
Relationship between initial plasma endothelin 1 concentrations and serum albumin concentrations following monthly monitoring of the focal and segmental glomerulosclerosis cohort (*p* = 0.03; r = −0.38). Specific patients are shown as red dots. The line indicating the trend is depicted by the red dotted line.

We also found a negative relationship between initial plasma endothelin 1 concentrations and initial serum albumin concentrations (*n* = 30; *p* = 0.05; r = −0.36) (Figure 19) after 1 month of monitoring in the focal and segmental glomerulosclerosis cohort (*n* = 30; *p* = 0.03; r = −0.38) (Figure 20).

**Figure 21:**
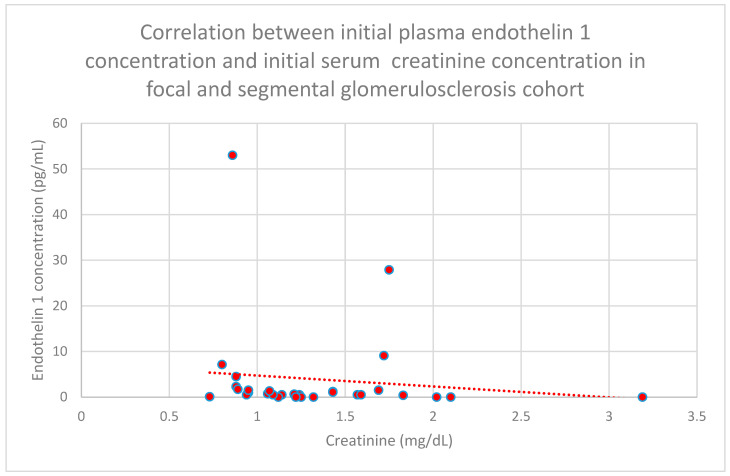
Relationship between initial plasma endothelin 1 concentrations and initial serum creatinine concentrations in the focal and segmental glomerulonephritis cohort (*p* = 0.03; r = −0.38). Specific patients are shown as red dots. The line indicating the trend is depicted by the red dotted line.

**Figure 22:**
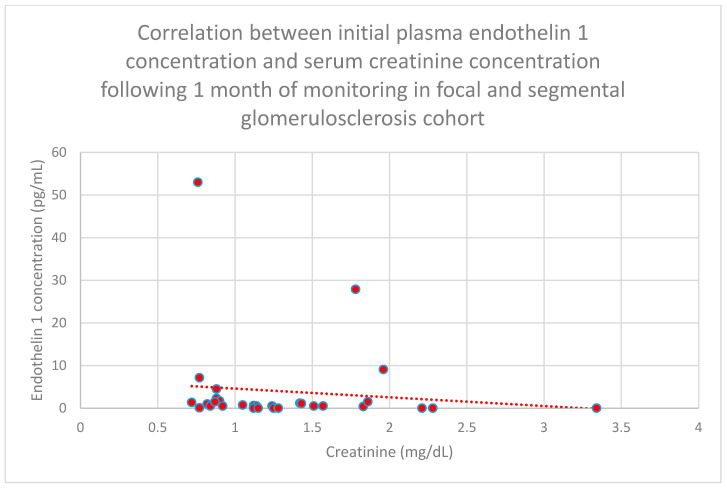
Relationship between initial plasma endothelin 1 concentrations and serum creatinine concentrations following 1 month of tracking in the focal and segmental glomerulonephritis cohort (*p* = 0.05; r = −0.36). Specific persons are shown as red dots. The line indicating the trend is depicted by the red dotted line.

Correlations between initial plasma endothelin 1 concentrations and initial serum creatinine concentrations (*n* = 30; *p* = 0.03; r = −0.38) (Figure 21) and serum creatinine concentrations following 1 month of monitoring (*n* = 30; *p* = 0.05; r = −0.36) (Figure 22) in the focal and segmental glomerulosclerosis cohort were statistically significantly negative.

**Figure 23:**
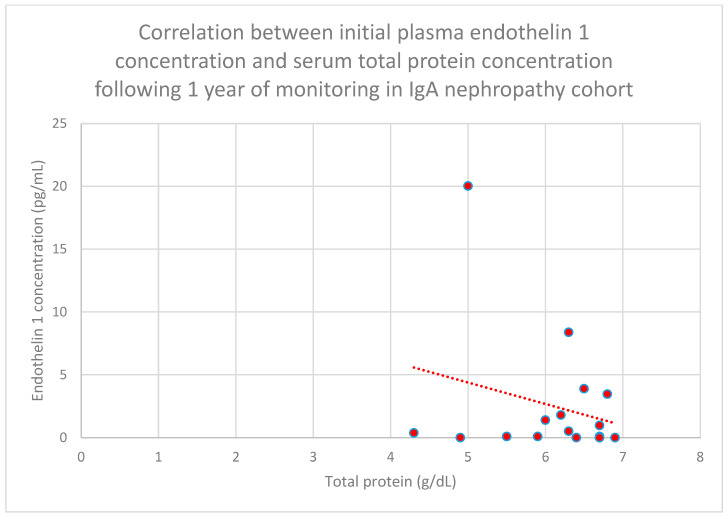
Relationship between initial plasma endothelin 1 concentrations and serum total protein concentrations following 1 year of monitoring in the IgA nephropathy cohort (*p* = 0.01; r = −0.61). Individual patients are shown as red dots. The line indicating the trend is depicted by the red dotted line.

The authors also found a statistically significant relationship between initial plasma endothelin 1 concentrations and total serum protein concentrations following 1 year of monitoring in the IgA nephropathy cohort (*n* = 16; *p* = 0.01; r = −0.61) (Figure 23).

**Figure 24:**
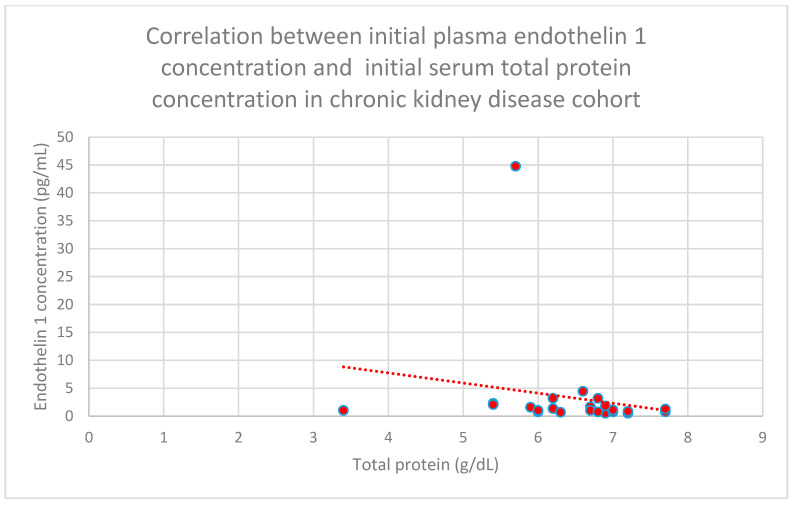
Relationship between initial plasma endothelin 1 concentrations and initial serum total protein concentrations in the chronic kidney disease cohort (*p* = 0.04; r = −0.43). Individual patients are shown as red dots. The line indicating the trend is depicted by the red dotted line.

**Figure 25:**
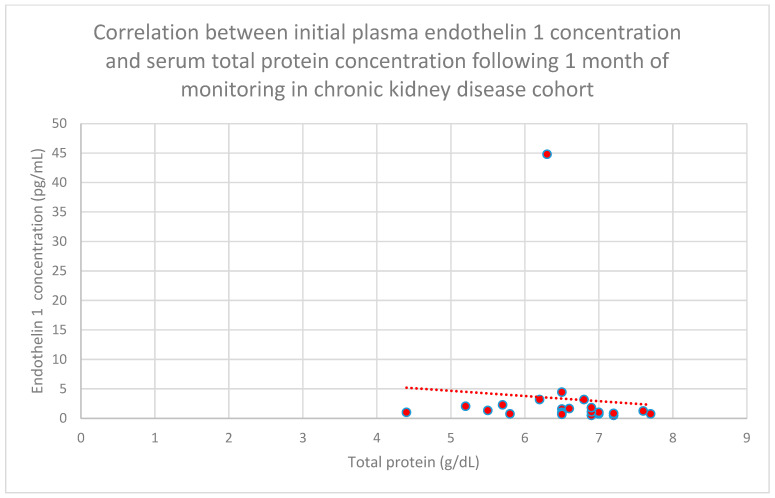
Relationship between initial plasma endothelin 1 concentrations and serum total protein concentrations following 1 month of monitoring in the chronic kidney disease cohort (*p* = 0.04; r = −0.43). Individual patients are shown as red dots. The line indicating the trend is depicted by the red dotted line.

**Figure 26:**
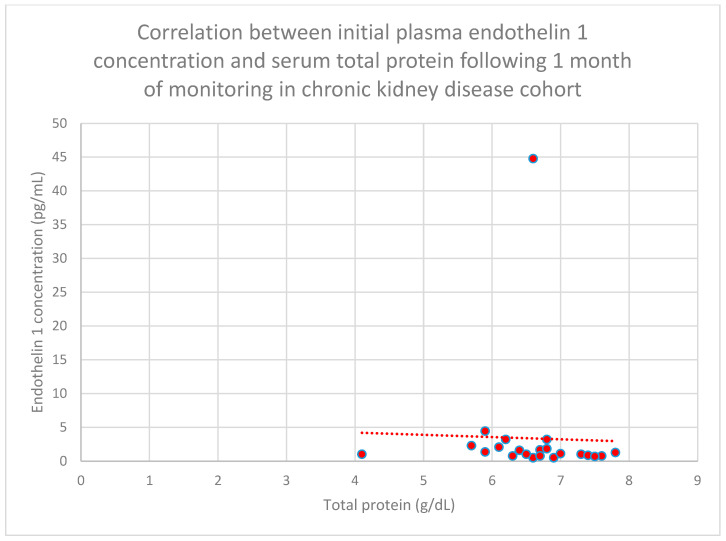
Relationship between initial plasma endothelin 1 concentrations and serum total protein concentrations following 1 year of monitoring in the chronic kidney disease cohort (*p* = 0.05; r = −0.4). Individual patients are shown as red dots. The line indicating the trend is depicted by the red dotted line.

Another relationship that was discovered is a correlation between initial plasma endothelin 1 concentrations and initial serum total protein concentrations in the chronic kidney disease cohort (*n* = 27; *p* = 0.04; r = −0.43) (Figure 24). This correlation was also statistically significant after 1 month (*n* = 27; *p* = 0.04; r = −0.43) (Figure 25) and 1 year of monitoring in the chronic kidney disease cohort (*n* = 27; *p* = 0.05; r = −0.4) (Figure 26).

**Figure 27:**
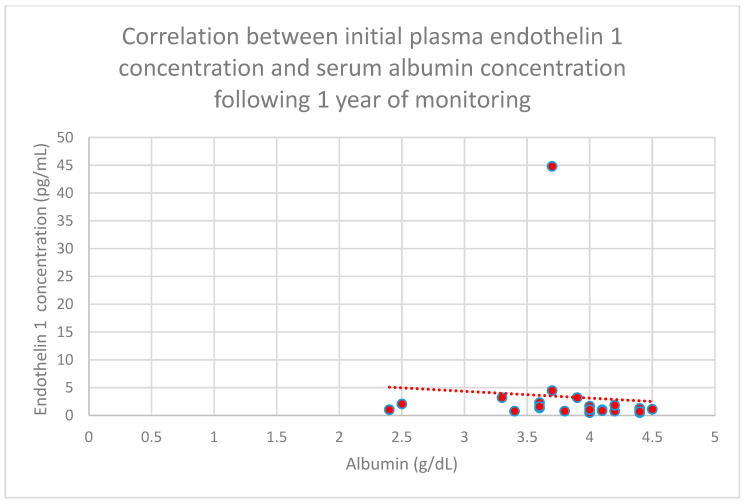
Relationship between initial plasma endothelin 1 concentrations and serum albumin concentrations following 1 year of monitoring in the chronic kidney disease cohort (*p* = 0.05; r = −0.4). Individual patients are shown as red dots. The line indicating the trend is depicted by the red dotted line.

**Figure 28:**
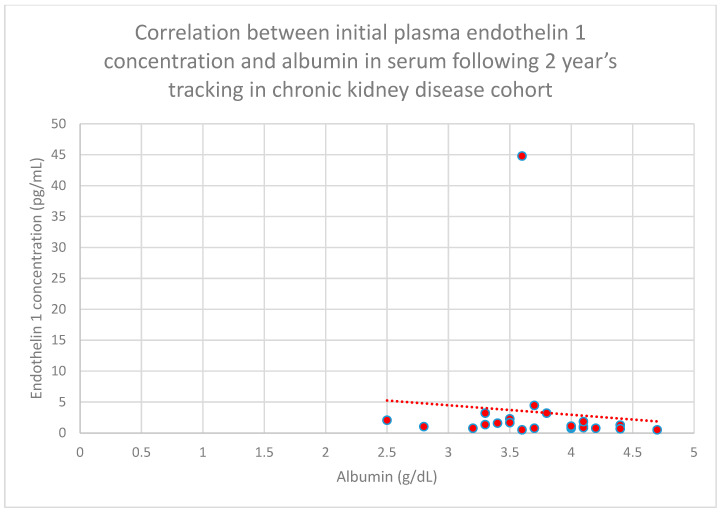
Relationship between initial plasma endothelin 1 concentrations and serum albumin concentrations following 2 years of tracking in the chronic kidney disease cohort (*p* = 0.05; r = −0.4). Individual patients are shown as red dots. The line indicating the trend is depicted by the red dotted line.

We also found a relationship between initial plasma endothelin 1 concentrations and serum albumin concentrations following 1 year (*n* = 27; *p* = 0.05; r = −0.4) (Figure 27) and 2 years (*n* = 27; *p* = 0.05; r = −0.4) (Figure 28) of monitoring in the chronic kidney disease cohort.

### 3.6. Correlations Between PAR 1 and Endothelin 1

**Figure 29:**
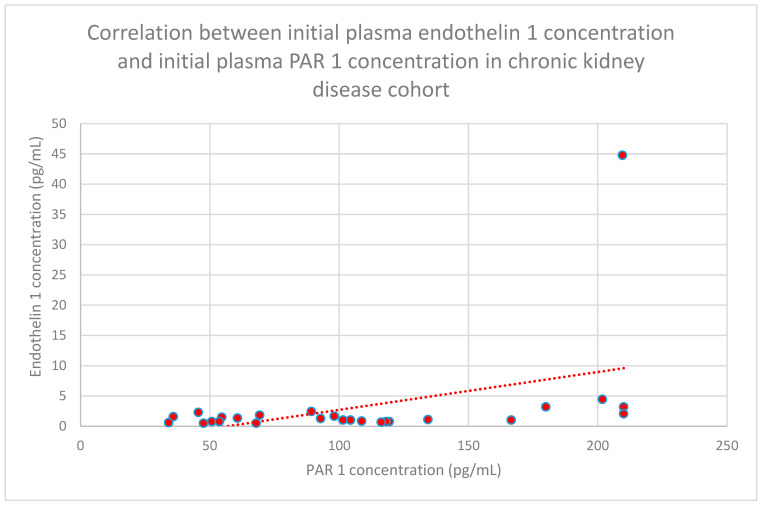
Relationship between initial plasma endothelin 1 concentrations and initial plasma PAR 1 concentrations in the chronic kidney disease cohort (*p* = 0.03; r = 0.41). Individual patients are shown as red dots. The line indicating the trend is depicted by the red dotted line.

The relationship between initial plasma endothelin 1 concentrations and initial plasma PAR 1 concentrations in the chronic kidney disease cohort was statistically significant (*n* = 27; *p* = 0.03; r = 0.41) (Figure 29).

### 3.7. Correlations Between PAR 1 and Anti-PAR1, PAR 1 and Anti-ETAR, Endothelin1 and Anti-PAR 1, Endothelin1 and Anti-ETAR, and Anti-PAR 1 and Anti-ETAR

We performed an analysis of the correlations between PAR 1, endothelin 1, the anti-PAR 1 antibody, and the anti-ETAR antibody in specific groups of patients.

**Figure 30:**
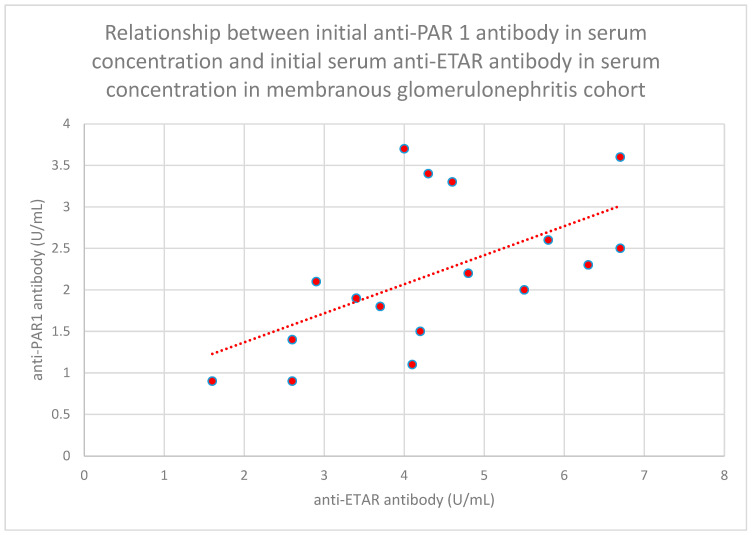
Relationship between initial serum anti-PAR 1 antibody concentrations and initial serum anti-ETAR antibody concentrations in the membranous nephropathy cohort (*p* = 0.004; r = 0.64). Individual patients are shown as red dots. The line indicating the trend is depicted by the red dotted line.

**Figure 31:**
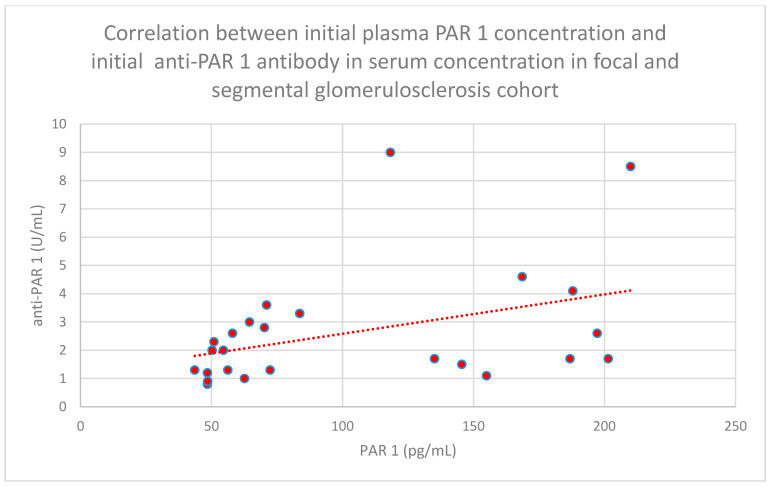
Relationship between initial plasma PAR 1 concentrations and initial serum anti-PAR 1 antibody concentrations in the focal and segmental glomerulonephritis cohort (*p* = 0.01; r = 0.48). Individual patients are shown as red dots. The line indicating the trend is depicted by the red dotted line.

**Figure 32:**
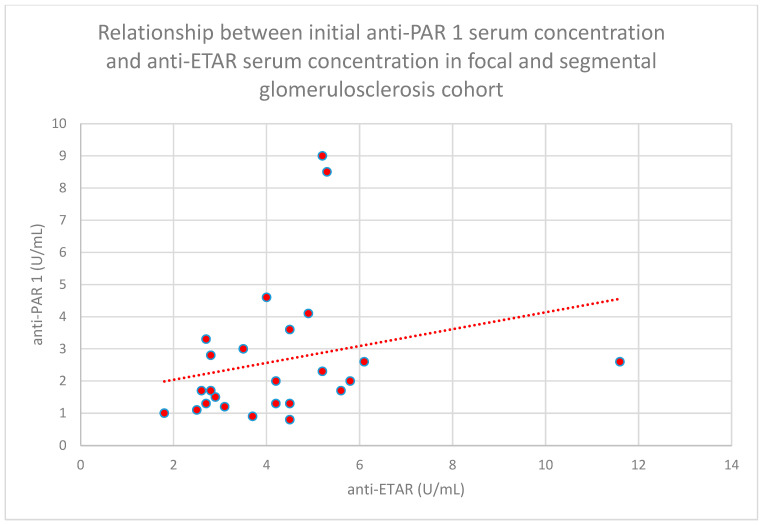
Relationship between initial serum anti-PAR 1 antibody concentrations and initial serum anti-ETAR antibody concentrations in the focal and segmental glomerulonephritis cohort (*p* = 0.04; r = 0.39). Individual persons are shown as red dots. The trend is represented by the red dotted line.

**Figure 33:**
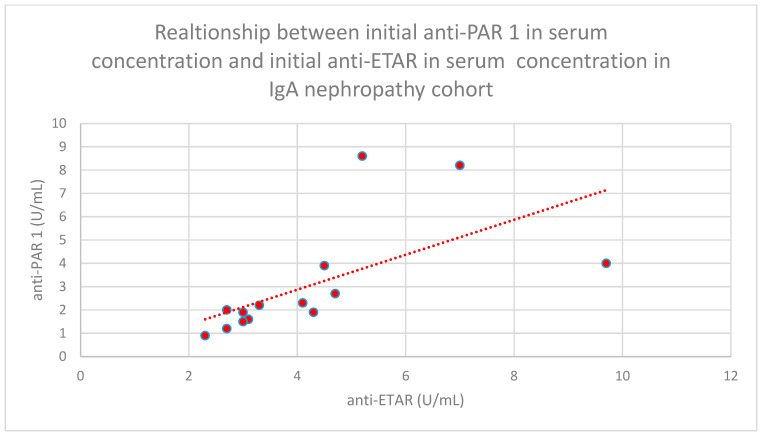
Relationship between initial serum anti-PAR 1 antibody concentrations and initial serum anti-ETAR antibody concentrations in the IgA nephropathy cohort (*p* < 0.001; r = 0.88). Individual patients are shown as red dots. The line indicating the trend is depicted by the red dotted line.

We found some statistically significant associations:

- The relationship between initial serum anti-PAR 1 antibody concentrations and initial serum anti-ETAR antibody concentrations in the membranous nephropathy cohort (*n* = 17; *p* = 0.004; r = 0.64) (Figure 30);
- The relationship between initial plasma PAR 1 concentrations and initial serum anti-PAR 1 antibody concentrations in the focal and segmental glomerulosclerosis cohort (*n* = 25; *p* = 0.01; r = 0.48) (Figure 31);
- The relationship between initial serum anti-PAR 1 antibody concentrations and initial serum anti-ETAR antibody concentrations in the focal and segmental glomerulosclerosis cohort (*n* = 25; *p* = 0.04; r = 0.39) (Figure 32);
- The relationship between initial serum anti-PAR 1 antibody concentrations and initial serum anti-ETAR antibody concentrations in the IgA nephropathy cohort (*n* = 14; *p* < 0.001; r = 0.88) (Figure 33).

## 4. Discussion

### 4.1. PAR 1 Levels

We found that levels of PAR 1 were higher in the FSGS, IgA nephropathy, chronic kidney disease, and hemodialysis groups compared to the healthy control group.

Higher levels of PAR 1 in FSGS support the results of the authors, who found that high PAR 1 levels are associated with a disease similar to FSGS in mice [27]. Anti-PAR 1 antibody levels were lower in the FSGS group compared to the healthy control group in the study that we performed previously. Moreover, the anti-PAR 1 antibody correlated positively with total protein levels, suggesting a stronger correlation with disease alleviation [16].

However, we did not find significant correlations between PAR 1 and clinical progress markers of focal and segmental glomerulosclerosis this time. This may be because of the small group size or a more complicated interplay between PAR 1 and the progression of FSGS. Some authors suggest that both an increase and a decrease in PAR 1 may be associated with the worsening of kidney function. Intermediate values of PAR 1 preserve kidney function the most optimally [28].

The PAR 1 levels observed in our study’s control group were comparable to those in the healthy control group from the other study [51]. The difference is that we used plasma in all groups of our study. The above-mentioned authors and other authors most commonly use serum. Plasma was used because we would like to analyze correlations between PAR 1 and endothelin 1 with anti-ETAR and anti-PAR 1 antibodies in the same samples collected from the same venepuncture, at the same time and from the same patients. That was the only material that we had after previous projects with which to perform such analyses. Similar results indicate that plasma may also be used as a source of materials for studies on PAR 1. The tests we used were validated for both serum and plasma.

We discovered a statistically significant positive correlation between PAR 1 levels and initial total protein levels, as well as albumin in SLE. Given that SLE is a highly active immunological disorder, such an association is definitely possible [52]. One may suspect that higher PAR 1 levels could be connected with decreased disease activity, although this correlation includes only initial total protein and albumin levels. This fact suggests that PAR 1 should not be used as a prognostic marker in SLE, but eventually only as a marker of actual disease activity. Interestingly, in our previous study on anti-PAR 1 correlations between the levels of this antibody and total protein levels, albumin levels were negative at many time points [16]. Data from both studies are consistent, strengthening the probability that these associations are true. PAR 1 levels correlated negatively with age in the SLE group. We supposed that it could be connected with diminished activity of the immunological system, which is associated with age [53].

We also found higher levels of PAR 1 in IgA nephropathy compared to the control group. PAR 1 levels correlated negatively with total protein level after 1 year of monitoring. Interestingly, in our previous study, the anti-PAR 1 antibody correlated negatively with creatinine after 2 years of monitoring [16]. These results suggest a possible role of PAR 1 in IgA nephropathy, but they are too sparse to be interpreted more seriously. These observations require confirmation in larger groups of patients.

PAR 1 levels were also higher in the chronic kidney disease and hemodialysis groups compared to the healthy control group. Moreover, in the chronic kidney disease cohort, initial PAR 1 was correlated with creatinine, with many time points of progressive monitoring. It is an open question whether PAR 1 relates to chronic kidney disease progression as a biomarker of a pathogenic process, or rather, chronic kidney disease’s influence on PAR 1 expression and metabolism. Mechanistic studies, such as in vitro or animal model studies, should be performed to explore the causality of this finding. 

### 4.2. Endothelin 1 Levels

Endothelin 1 levels in specific groups were not different compared to the control group. Nevertheless, we found many statistically significant correlations between endothelin 1 levels and clinical markers of specific disease progression.

Basic endothelin 1 levels correlated negatively with albumin at many monitoring time points in the membranous nephropathy group. This observation suggests that endothelin 1 could be used as a possible predictor of albumin levels in membranous nephropathy.

Initial plasma endothelin 1 concentrations in the cohort of patients with focal and segmental glomerulosclerosis correlated negatively with both total protein and albumin levels at many time points during the follow-up period, so higher levels of endothelin 1 were associated with worse progression of the disease in terms of albumin and total protein levels. Simultaneously, endothelin 1 showed a negative correlation with creatinine initially and after a short time of observation. Together, our data indicate that the influence of endothelin 1 on the progression of FSGS is complex. There are many factors that can influence the course of the disease in this way. An example is angiotensin II-converting enzyme inhibitors. This group of drugs diminishes proteinuria and increases total protein and albumin, but these drugs may diminish blood flow through the kidneys and, as a result, cause an elevation in creatinine [54]. Moreover, correlations between endothelin 1 and total protein, and albumin and creatinine, in the focal and segmental glomerulosclerosis cohort are weak (r = 0.36–0.4)—weaker than the other correlations presented in this study, despite the fact that the FSGS group was the most populous of all the groups. Many statistically important correlations of endothelin 1 are connected with the lack of difference between the level of endothelin 1 in the control group and specific kidney diseases, suggesting that endothelin 1 may act as a modulator of kidney disease progression rather than as a biomarker connected with the pathogenesis of specific diseases.

Our patients were treated during the monitoring period, so the treatment might have had an influence on these results. Interestingly, in our previous study, the FSGS cohort displayed anti-ETAR antibody levels that were lower than the healthy control group’s [17]. Although we were not able to find any important correlations with the time progression of FSGS in that study, data from both studies suggest some kind of disequilibrium between endothelin 1 and anti-ETAR in FSGS patients. It is possible that, in a study involving larger groups of patients, more statistically important data could be revealed. Our data are confirmed by the data from the DUET study, where sparsentan (a simultaneous endothelin A receptor and angiotensin II type 1 receptor antagonist) effectively diminished proteinuria [48].

Initial plasma levels of endothelin 1 correlated negatively with creatinine after 1 year of monitoring in IgA nephropathy. This is only a single correlation, but it is consistent with the fact that the anti-ETAR antibody from our previous study correlated positively with creatinine after 2 years of follow-up [17]. Data from the ALIGN trial, where the endothelin A receptor antagonist atrasentan effectively reduced proteinuria [50], were similar to the project where the molecular expression of endothelin 1 was correlated with the risk of IgA nephropathy progression [38], so we think that our observed endothelin 1 correlation in this disease is connected with the results of the treatment, not real pathogenic effects.

Endothelin 1 also correlated negatively with total protein and albumin levels at many different time points during the follow-up period in the chronic kidney disease cohort, suggesting that it could be a predictor of total protein and albumin levels in this group of patients. This finding was earlier postulated by other authors [55].

### 4.3. Correlations Between PAR 1 and Endothelin 1

PAR 1 and endothelin 1 were correlated, although only in the chronic kidney disease group. These observations suggest that chronic kidney disease has a similar influence on the metabolism of these markers. PAR 1 correlated with creatinine in our observations, and endothelin 1 correlated negatively with total protein and albumin in this group. A real pathogenic connection between these receptors in chronic kidney disease is not definitively excluded, although it is difficult to track because of the heterogeneity of this group in terms of the different diseases that cause chronic kidney disease. Moreover, studies on molecular models should be performed to find out what was first: chronic kidney disease’s influence on PAR 1 and endothelin 1, or whether these biomarkers modulate CKD (chronic kidney disease).

### 4.4. PAR 1, Endothelin 1, Anti-ETAR, and Anti-PAR 1 Antibody Correlations

We also performed a correlation analysis between PAR 1, endothelin 1, anti-ETAR, and the anti-PAR 1 antibody. We had data about the antibody levels from our previous studies: anti-PAR 1 [16] and anti-ETAR [17]. Some of the patients with membranous nephropathy, FSGS, lupus nephritis, IgA nephropathy, and mesangial (non-IgA) proliferative glomerulonephritis recruited for that study were the same patients recruited for this study, and materials were taken from the same patient, on the same day, and during the same venepuncture. Anti-PAR 1 and anti-ETAR antibodies were correlated in the membranous nephropathy, focal and segmental glomerulosclerosis, and IgA nephropathy cohorts, suggesting that factors causing the induction of the expression of these antibodies can influence both kinds of antibodies.

PAR 1 and the anti-PAR 1 antibody were correlated in the FSGS group, giving additional confirmation to the earlier published studies [16,27,48], which showed that PAR 1 is probably involved in FSGS pathogenesis. Moreover, it can be supposed that an equilibrium between PAR 1 and anti-PAR1 could influence the development of these diseases. PAR 1 changes in both directions: both an increase and a decrease could deteriorate kidney function [28], so the mechanism of anti-PAR1 production could theoretically be a natural reaction of the organism for PAR 1 level elevation to maintain homeostasis.

### 4.5. Study Limitations

Individuals diagnosed with FSGS, membranous nephropathy, and mesangial proliferative (non-IgA) glomerulopathy were provided with conventional immunosuppressive treatment throughout the study duration. This regimen consisted of administering three doses of methylprednisolone, each 500 mg, followed by a course of oral prednisone at a rate of 1 mg/kg with a gradual tapering of the dosage. Patients suffering from IgA nephropathy received methylprednisolone (500 mg) on three occasions, each two months apart. Additionally, for a period of 6 months, prednisone (0.5 mg/kg) was administered every alternate day to the patients, between the doses of methylprednisolone. Azathioprine (100 mg) was administered daily, whereas cyclophosphamide (500 mg) was provided biweekly for a total of six doses, along with an initial dose of prednisone (1 mg/kg of body weight), which was incrementally reduced and included as a component of the therapy for lupus nephritis.

Basic biomarkers and clinical parameters were not influenced by the treatment because, at the time of the initial material collection, none of the patients were receiving any treatment. The clinical results after a period of observation may be influenced by the treatment. Effective treatment may decrease creatinine levels or increase total protein and albumin levels. The results were not adjusted for the influence of the treatment, because the groups were too small, and also due to the above-mentioned fact that treatment changes progress over time according to the above-mentioned regimens. Dividing our groups into many subgroups would be statistically unacceptable, as the subgroups would be too small.

Nevertheless, the directions of the correlations (positive or negative) were consequently the same in specific diseases. This phenomenon contradicts the hypothesis about the accidental values of the results. The answer to this question is that the correlations are so strong that even treatment barely influences them, although the question of whether the correlations are the result of the treatment would require another study on larger groups of patients in the future. Several disease groups have small sample sizes. This constrains the applicability of the results and heightens the likelihood of type II errors, so our data are preliminary, and these results should be checked in larger groups of patients. As we tested numerous correlations across diseases and multiple time points, there is a high risk of type I errors, although we adjusted our results for multiple comparisons with the Bonferroni method. Only the initial levels of PAR 1 and endothelin 1 were evaluated. Variations in these biomarkers as time progressed could offer improved prognostic significance. We suggest that upcoming research should incorporate repeated measurements to evaluate the dynamics of biomarkers.

### 4.6. Study Strengths

On the other hand, we detected many consistent associations that were statistically significant despite the specific cohorts not being very numerous. This phenomenon suggests the real character of the observed relationships, especially as they were consistent with observations from our previous studies [16,17]. Our study uses a multicohort design, including healthy controls. We collected longitudinal clinical data over 2 years. We integrated this study with prior antibody studies (anti-PAR 1 and anti-ETAR).

Our study is the first worldwide evaluation of PAR 1 in specific kidney diseases, and we are the first to find correlations between PAR 1 and anti-PAR 1 in FSGS and correlations between anti-PAR 1 and anti-ETAR in membranous nephropathy, FSGS, and IgA nephropathy.

### 4.7. Future Perspectives

The findings of this study require validation with a more extensive sample (studies with larger cohorts and serial sampling of PAR1 and endothelin 1 at different time points), and should be further evaluated through a prospective cohort framework. Furthermore, additional investigations are necessary to explore the activity of PAR 1 and endothelin 1 in specific glomerular conditions, utilizing molecular models and mechanistic studies to reveal the real causality of the observed associations. Future projects ought to encompass a meta-analysis of current research and artificial intelligence models to attain a thorough comprehension of the interactions among various markers and antibodies in glomerular disorders. The possibility of treating some diseases with the usage of ETAR inhibitors means that this area is an interesting topic to explore.

Regardless of whether the PAR 1 correlations were a result of CKD, or are connected with pathogenetic processes, this biomarker seem to be connected with the progress of CKD and could serve as a prognostic marker for CKD.

## 5. Conclusions

PAR 1 levels are elevated in membranous nephropathy, focal and segmental glomerulosclerosis, IgA nephropathy, chronic kidney disease, and hemodialysis patients compared to healthy controls.

PAR 1 correlates with markers of clinical progress in SLE and chronic kidney disease cohorts.

We supposed that PAR 1 may serve as a prognostic marker in CKD.

PAR 1 correlates with anti-PAR 1 in FSGS.

Endothelin 1 was supposed to be related to the clinical course of membranous nephropathy, FSGS, IgA nephropathy, and chronic kidney disease.

Anti-PAR 1 correlates with anti-ETAR in membranous nephropathy, FSGS, and IgA nephropathy.

## Figures and Tables

**Table 1:** Clinical data (median values) of persons from the evaluated cohorts.

Specific Kidney Disease	Initial Level of Creatinine in Serum (mg/dL)	Initial Estimated Glomerular Filtration Rate (mL/min./ 1.73 m^2^) MDRD	BUN (mg/dL)	Albumin/Creatinine Ratio	Amount of Protein in Urine (g/per Day)	Initial Total Protein Level in Serum (g/dL)	Initial Albumin Level in Serum (g/dL)
membranous glomerulonephritis (*n* = 19)	1.17 (0.61–3.3)	70 (15–116)	11 (8–32)	1.52 (0.06–7.34)	2.64 (1.13–5.71)	4.9 (4.35–5.6)	2.8 (2.5–3.2)
focal and segmental glomerulosclerosis (*n* = 30)	1.22 (0.73–3.19)	62 (31–126)	11 (5–30)	1.08 (0.3–7.5)	2.14 (0.04–7.46)	5.35 (4.4–6.1)	3.2 (2.5–3.6)
systemic lupus erythematosus (*n* = 22)	1.10 (0.74–2.19)	60 (24–116)	9 (4–23)	0.71 (0.02–3.13)	1.26 (0.27–2.24)	5.7 (5.2–6.4)	3.3 (3–3.9)
IgA nephropathy (*n* = 16)	0.92 (0.59–1.55)	68 (35–131)	9.5 (6–20)	0.6 (0.05–2.2)	1.06 (0.64–2)	5.65 (4.9–6.3)	3.4 (2.75–4)
mesangial proliferative (non-IgA) glomerulonephritis (*n* = 7)	0.91 (0.59–1.55)	90 (40–131)	9 (6–16)	0.82 (0.17–3.4)	1.46 (1.14–4.88)	5.0 (4.5–5.2)	2.8 (2.3–3.2)
control group (*n* = 22)	0.94 (0.68–1.19)	70 (64.3–101.6)	9 (7–11)	0(0–0)	0(0–0)	7.3 (6.5–8.3)	4.5 (4.2–5.2)
chronic kidney disease (*n* = 27)	2.69 (1.23–10.51)	23 (5–52)	25.5 (11–97)	0.42 (0.04–12.2)	0.82 (0.08–23.8)	6.7 (3.4–7.7)	3.8 (2–4.6)
hemodialysis (*n* = 26)	5.2 (3.2–8.4)	11 (6–18)		Not applicable	Not applicable	6.4 (4.5–7.6)	3.65 (2.8–4.3)

Legend: The numbers displayed in the table represent median values accompanied by their respective clinical data ranges (which are provided in parentheses) across designated cohorts.

**Table 2:** Clinical data (median values) of persons from evaluated cohorts.

Specific Kidney Disease	Age (Years)	Sex (Percent of Males)	Hb (g/dL)	Hct (%)	Leukocytes (Number/Microliter)
membranous glomerulonephritis (*n* = 19)	50.5 (39–60.5)	80%	13.4 (11.5–16)	45 (37.2–52.6)	6.8 (2.5–10.8)
focal and segmental glomerulosclerosis (*n* = 30)	47 (31–59)	67%	13.6 (9.3–16.8)	44.6 (30.6–55)	7.9 (4.1–11)
lupus nephritis (*n* = 22)	34.5 (31–47)	27%	13 (10.5–17.3)	42.6 (34.5–59)	6.3 (2.9–10.9)
IgA nephropathy (*n* = 16)	45.5 (31–59)	50%	14.6 (12.2–16.4)	48.1 (40.1–54)	8.4 (4.4–10.9)
Mesangial proliferative (non-IgA) glomerulonephritis (*n* = 7)	22 (20–52)	28%	14.5 (10.1–18)	47.5 (33.2–55)	8.1 (6–10.5)
control group (*n* = 22)	47.5 (30–59)	54%	14.5 (9.4–17.9)	43.4 (37–50)	6.3 (3.5–8.3)
chronic kidney disease (*n* = 27)	47 (19–64)	59%	12.9 (8.2–18.1)	38.8 (25–51.4)	8.1 (4.4–11)
hemodialysis (*n* = 26)	47.5 (36–62)	54%	10 (7.4–12.8)	30.2 (23–41)	6.9 (4.4–11)

Legend: The numbers displayed in the table represent median values accompanied by their respective ranges (which are provided in parentheses) of clinical parameters across designated groups. Hb—hemoglobin; Hct—hematocrit.

## Data Availability

The article, together with the Supplementary Materials, encompasses the data and the original contributions presented by this study. Should you have any additional inquiries, please reach out to the corresponding author.

## Supplementary Materials

The additional supplementary information can be found at: https://www.mdpi.com/article/10.3390/jcm15010221/s1, Figure S1: Key: 1-membranous nephropathy; 2-focal and segmental glomerulosclerosis; 3systemic lupus erythematosus; 4-IgA nephropathy; 5-mesangial (non-IgA) proliferative glomerulonephritis; 6-hemodialysis; 7-chronic kidney disease; 8-control group. Figure S2: Key: 1-membranous nephropathy; 2-focal and segmental glomerulosclerosis; 3 systemic lu-pus erythematosus; 4-IgA nephropathy; 5-mesangial (non-IgA) proliferative glomerulonephritis; 6-hemodialysis; 7-chronic kidney disease; 8-control group.

## Abbreviations

ANAs	antinuclear antibodies
ANOVA	analysis of variance
BUN	blood urea nitrogen
CKD	chronic kidney disease
CRP	C-reactive protein
dsDNA	anti-double-stranded deoxyribonucleic acid antibodies
eGFR	estimated glomerular filtration
ELISA	enzyme-linked immunosorbent assay
ETAR	endothelin A receptor
ETBR	endothelin B receptor
FSGS	focal and segmental glomerulosclerosis
Hb	hemoglobin
Hct	hematocrit
IgA	immunoglobulin A
IL	interleukin
IU	international unit
NF	nuclear factor
PAR 1	protease activated receptor
SLE	systemic lupus erythematosus
TGF	transforming growth factor
TNF	tumor necrosis factor
U	unit
