# Supplementary figures and images for: The Influence of PAR 1 and Endothelin 1 on the Course of Specific Kidney Diseases
Source: J Clin Med. 2025 Dec 27;15(1):221. doi: 10.3390/jcm15010221 (PMC12786784; doi:10.3390/jcm15010221)

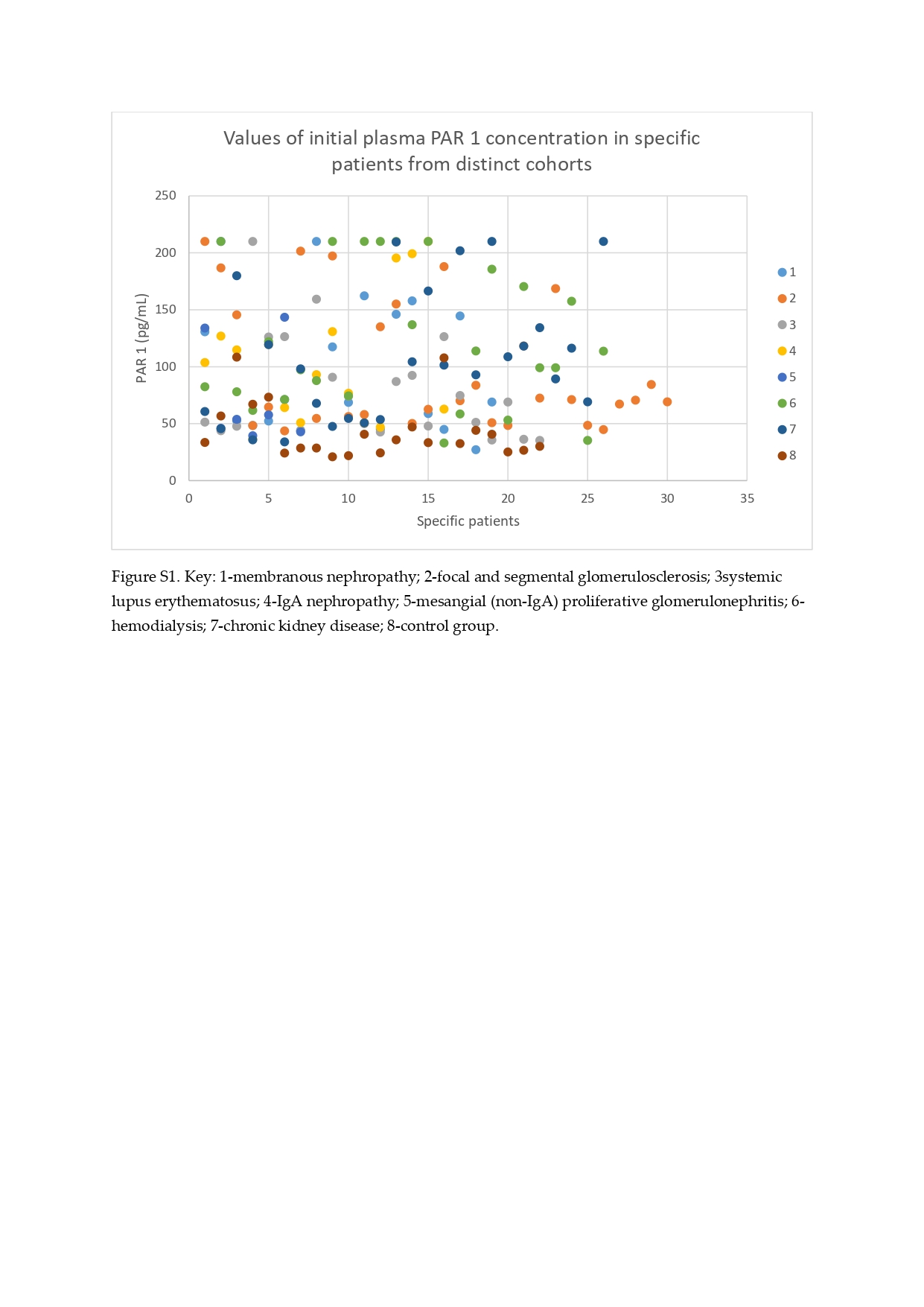

Supplement: Supplementary file 1 [file jcm-15-00221-s001.zip › Figure S1.jpg]

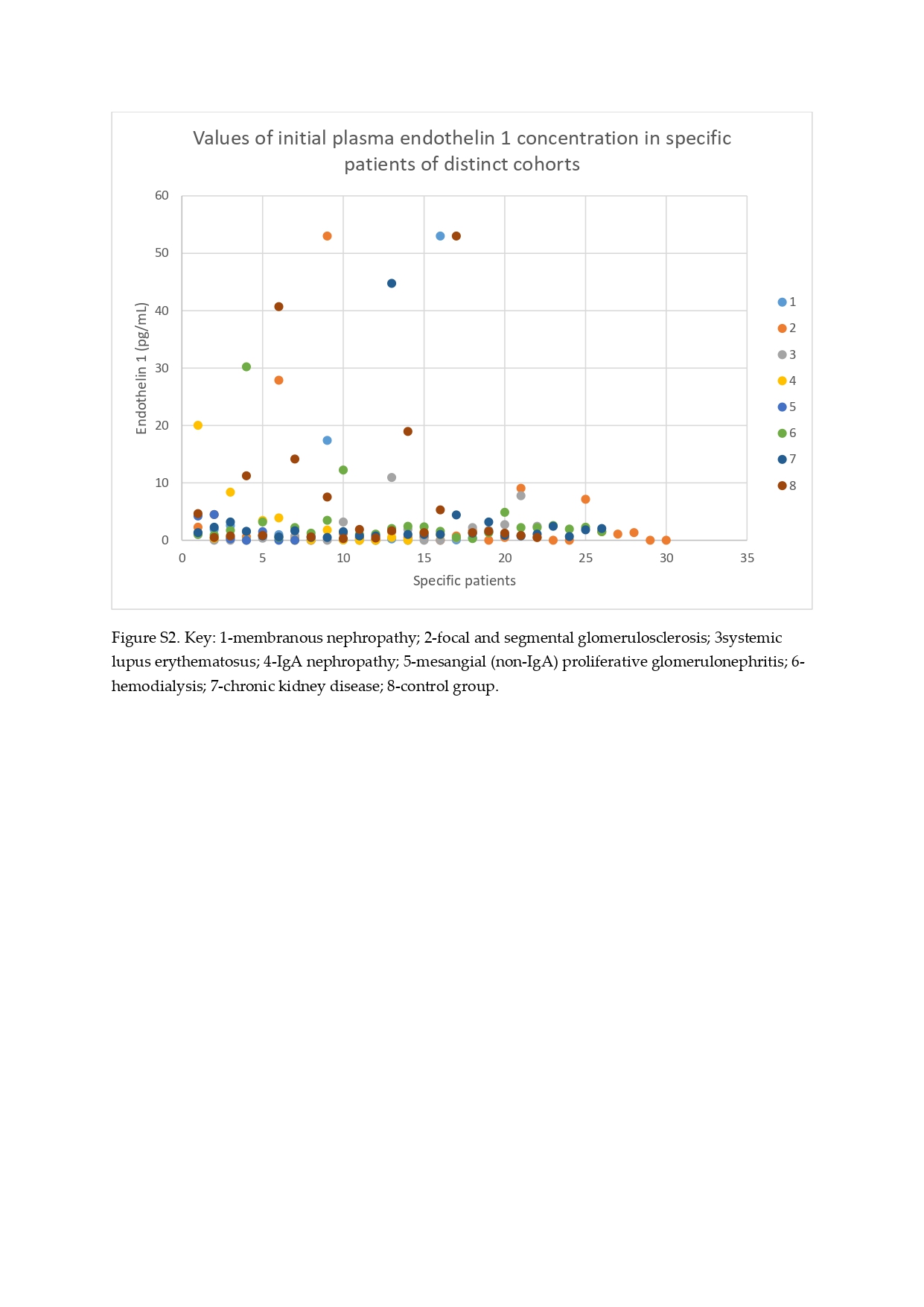

Supplement: Supplementary file 1 [file jcm-15-00221-s001.zip › Figure S2.jpg]
